# Clinical and molecular insights into cardiovascular disease in psoriatic patients and the potential protective role of apremilast

**DOI:** 10.3389/fimmu.2024.1459185

**Published:** 2024-08-07

**Authors:** Nuria Barbarroja, Clementina López-Medina, Alejandro Escudero-Contreras, Iván Arias-de la Rosa

**Affiliations:** Rheumatology Service, Department of Medical and Surgical Sciences, Maimonides Institute for Research in Biomedicine of Cordoba (IMIBIC), University of Cordoba, Reina Sofia University Hospital, Córdoba, Spain

**Keywords:** apremilast, cardiometabolic comorbidities, cardiovascular disease, psoriatic arthritis, psoriasis, psoriatic disease

## Abstract

Psoriatic disease, encompassing both psoriasis (Pso) and psoriatic arthritis (PsA), is closely intertwined with a significantly elevated risk of developing cardiovascular diseases. This connection is further compounded by a higher prevalence of cardiometabolic comorbidities, including type 2 diabetes, obesity, insulin resistance, arterial hypertension, and dysregulated lipid profiles. These comorbidities exceed the rates seen in the general population and compound the potential for increased mortality among those living with this condition. Recognizing the heightened cardiometabolic risk inherent in psoriatic disease necessitates a fundamental shift in the treatment paradigm. It is no longer sufficient to focus solely on mitigating inflammation. Instead, there is an urgent need to address and effectively manage the metabolic parameters that have a substantial impact on cardiovascular health. Within this context, apremilast emerges as a pivotal treatment option for psoriatic disease. What sets apremilast apart is its dual-action potential, addressing not only inflammation but also the critical metabolic parameters. This comprehensive treatment approach opens up new opportunities to improve the well-being of people living with psoriatic disease. This review delves into the multifaceted aspects involved in the development of cardiovascular disease and its intricate association with psoriatic disease. We then provide an in-depth exploration of the pleiotropic effects of apremilast, highlighting its potential to simultaneously mitigate metabolic complications and inflammation in individuals affected by these conditions.

## 1 Introduction

Psoriasis (Pso), is a persistent inflammatory skin condition, exhibiting autoimmune pathogenic features and a heightened susceptibility to immune-mediated genetic factors. The prevalence of the disorder aligns with its degree of severity. While commonly known as psoriasis vulgaris, it is also denoted as plaque-type psoriasis, characterized by patchy manifestations on the extremities. The chronic plaque form prevails in around 90% of patients, manifesting clinical features such as well-defined, erythematous patches that are partially pruritic and covered in silvery scaling (1). Around 30% of patients with Pso will develop arthritis at any time over their lives. Individuals with Psoriatic Arthritis (PsA), categorized under the umbrella of Spondyloarthritis, often experience impaired function and reduced quality of life. Typically, psoriasis precedes arthritis by about 10 years, although in 20% of cases, both occur simultaneously or PsA develops first (2). Diagnosis relies on clinical and imaging features across five domains: psoriasis, peripheral joint disease, axial disease, enthesitis, and dactylitis. Rheumatoid factor tests are usually negative in 95% of PsA patients, while about 25% are HLA-B27-positive. Notably, PsA is characterized by bone and cartilage destruction and pathological new bone formation (3).

PsA and Pso have been extensively linked to various coexisting conditions, including obesity, type 2 diabetes, arterial hypertension, metabolic syndrome, fatty liver, and an increased risk of cardiovascular (CV) events (3–5). Multiple studies have evidenced the heightened prevalence of cardiovascular disease (CVD) risk factors in PsA when compared to Pso, indicating a potential contribution of inflammatory joint disease to cardiovascular morbidity (6). However, additional studies are imperative to establish definitively the extent of association between PsA, Pso, and CVD (7). On the other hand, we observed that patients with PsA have a higher prevalence of CVD comorbidities compared to those with other types of inflammatory arthritis. These comorbidities include an elevated ApoB/ApoA ratio, increased atherogenic risks, obesity, insulin resistance (IR), hyperlipidemia, arterial hypertension, and type 2 diabetes mellitus (8). Furthermore, there is a research gap concerning the impact of various treatments on CVD in Psoriatic Disease. Nevertheless, studies have provided support for the potential positive effect of Apremilast on CVD associated with PsA (9). This review aims to describe the relationship between CVD comorbidities and Psoriatic Disease and to evaluate the impact of Apremilast, a phosphodiesterase-4 inhibitor, from both clinical and molecular perspectives. To accomplish this, a rigorous selection process was implemented, which involved searching for original research articles and review publications written in English. The databases PubMed, Web of Science, and Scopus were utilized, using keywords such as “CVD”, “Cardiovascular Disease,” “Psoriatic Arthritis”, “Psoriasis”, “Psoriatic Disease”, “Apremilast”, or “anti-PDE4”.

## 2 Enhancers of cardiometabolic risk factors

### 2.1 Obesity

Obesity is conventionally characterized as the presence of excessive body fat that negatively impacts health. In clinical settings, it is typically evaluated using the body mass index (BMI= kg/m2) (10). Despite significant progress in combating CVD, obesity remains a modifiable risk factor that has not been adequately addressed. Lifestyle changes and pharmacotherapy have not effectively tackled obesity, unlike other risk factors such as hypertension, dyslipidemia, T2DM, and smoking. Beyond GLP-1 inhibitors for obesity management, pharmacotherapy has not successfully addressed this condition. Research suggests that the increased CVD risk associated with a high BMI or elevated waist circumference is largely mediated by changes in intermediate risk factors such as atherogenic dyslipidemia, hypertension, and T2DM (11). Numerous studies have examined the connection between obesity and adverse CV events (12). Physicians have effective medications targeting lipids, blood pressure, and glycemic control, backed by strong evidence from large trials. However, upstream causes like high-risk adiposity may not be prioritized as treatment targets. Addressing excessive adiposity, a major contributor to elevated lipids, blood pressure, and glucose levels, could substantially reduce CVD risk (13).

Moreover, Virtue and Vidal-Puig introduced a hypothesis positing that each individual might have an inherent limit to the expandability of their adipose tissue. As one gains weight, there reaches a point where their adipose tissue can no longer accommodate additional lipids. Beyond this threshold, there is an increased flux of lipids to non-adipose organs, initiating the deposition of ectopic lipids. The build-up of lipids in cells such as myocytes, hepatocytes, and beta cells subsequently trigger adverse effects, including IR and apoptosis, which can have detrimental effects on overall health (14). Considering these insights, it becomes evident that one can be obese without being IR. In alignment with this notion, our group demonstrated that individuals with obesity who do not exhibit IR do not manifest the inflammation levels on their adipose tissue typically associated with obese patients who do have IR. This suggests that the critical link between adipose tissue expansion and the accompanying metabolic complications may be the degree of inflammation in the adipose tissue (15). Taking into account this hypothesis, targeting “dysfunctional” adipose tissue might be the right way to reduce the collateral downside effects such as IR, diabetes, hypertension or dyslipidaemia.

### 2.2 Diabetes mellitus and hyperglycemia

Diabetes mellitus is a chronic condition characterized by persistently high blood glucose levels and is classified into two main subtypes: type 1 diabetes mellitus (T1DM) and type 2 diabetes mellitus (T2DM). T1DM is considered an autoimmune disease, wherein the body’s immune system attacks and destroys beta cells in the pancreas, leading to insulin deficiency. In contrast, the role of autoimmunity in the development of T2DM is not as well-defined and remains less clear (16). Moreover, atherosclerosis is a prevalent characteristic among individuals with diabetes mellitus and is responsible for severe macrovascular complications, including coronary artery disease (CAD), stroke, and peripheral vascular disease (17). Prior research has consistently associated patients with T1DM with elevated rates of coronary calcification and increased mortality ratios related to CAD (18, 19). On the other hand, T2DM continues to be associated with CVD as the primary cause of death globally. While there has been a decline in mortality due to aggressive treatments, the growing number of diabetic patients, especially among younger and elderly populations, raises significant concerns (20).

In T1DM, hyperglycemia typically develops rapidly without preceding IR. Over time, prolonged hyperglycemia in T1DM leads to an increase in IR (21). In contrast, T2DM manifests with IR for years before noticeable hyperglycemia, resulting in a gradual rise in blood sugar levels. The presence of IR in both T1DM and T2DM complicates isolating the effects of hyperglycemia alone on CVD and its risk factors (22). However, sustained hyperglycemia, regardless of diabetes type, is known to contribute to various complications, including cardiovascular issues.

### 2.3 Insulin resistance

IR occurs when insulin loses its normal effects in vital metabolic tissues such as adipose tissue, skeletal muscle, or liver. A significant meta-analysis involving 516,325 patients revealed that the homeostatic model assessment (HOMA) was a superior predictor of CVD events in adults without T2DM compared to fasting glucose or insulin (23). However, fasting insulin, a classic marker of IR, has also shown associations with CVD events in individuals without T2DM, independent of other risk factors (24). Notably, IR plays a critical role in promoting atherosclerosis, particularly at the arterial wall level. It affects macrophages and endothelial cells, contributing to both the initiation of atherosclerosis (atherogenesis) and the progression of clinically significant advanced plaques (25). Understanding the impact of IR on atherosclerosis can provide valuable insights for better managing cardiovascular health and preventing related complications.

### 2.4 Arterial hypertension

Hypertension, often referred to as systemic arterial hypertension, is a medical condition characterized by a persistent elevation in blood pressure (BP) within the systemic arteries. BP is typically expressed as a ratio of systolic BP (the pressure exerted on arterial walls during heart contractions) to diastolic BP (the pressure during heart relaxation) (26). This complex condition is influenced by various facets of the cardiovascular system, including blood volume, cardiac output (the volume of blood pumped by the heart per minute), and the complex balance of arterial tone, which is impacted by both intravascular volume and neurohormonal systems. The regulation of healthy BP levels involves a sophisticated interplay among components within an integrated neurohumoral system. This system encompasses the renin-angiotensin-aldosterone system, the roles played by natriuretic peptides and the endothelium, the sympathetic nervous system, and the immune system. Any malfunction or disruption of these factors, within any of these systems, can directly or indirectly lead to increases in mean BP, BP variability, or both, over time. Consequently, this can result in damage to target organs, such as left ventricular hypertrophy and chronic kidney disease, and contribute to adverse outcomes in CVD (27).

### 2.5 Dysregulated lipid profile and fatty liver disease

Dyslipidemias can be classified into two main categories: genetically determined types, referred to as primary or familial dyslipidemias, and those arising as secondary consequences of underlying conditions like diabetes mellitus, obesity, or an unhealthy lifestyle—this secondary group being more prevalent. Among these conditions, heightened plasma LDL-cholesterol levels emerge as significant risk factors for CVD. Dyslipidemias entail deviations from the normal plasma lipid profile, often linked to various clinical conditions. The most common form is hypercholesterolemia, characterized by elevated cholesterol levels, closely associated with an increased CVD risk. Furthermore, atherogenic dyslipidemia, characterized by elevated triglyceride levels, diminished HDL-cholesterol levels, and the presence of low-density lipoproteins (LDL) particles, is notably prevalent among patients with diabetes or metabolic syndrome. This particular dyslipidemia profile significantly heightens their CVD risk.

However, dyslipidemias manifest in diverse forms, including hypertriglyceridemia, associated with severe conditions such as non-alcoholic fatty liver disease (NAFLD) (28). Moreover, the interplay between CVD and hepatic damage extends beyond adipose tissue dysfunction. It encompasses various mechanisms, including the dysregulation of lipid metabolism, where the liver plays a pivotal role. Dyslipidemias associated with CVD and hepatic damage often involve altered hepatic lipid synthesis, uptake, and secretion. Additionally, NAFLD, a prevalent hepatic condition in the context of metabolic syndrome, further complicates this relationship. NAFLD not only contributes to dyslipidemia but also serves as a source of systemic inflammation, which can exacerbate CVD risk (29). This interplay is not limited to CVD and hepatic damage alone; it extends to a network of mediators that bridge the two systems. Inflammation and oxidative stress, for instance, act as crucial mediators in both cardiovascular diseases and liver disorders. These factors can initiate and perpetuate a cycle of damage, promoting systemic inflammation and vascular dysfunction. Additionally, the influence of hemodynamic changes, neurohormonal activation, and immune system responses further underscores the bidirectional relationship between these two vital organ systems. Understanding and managing these multifaceted interactions is crucial for effectively addressing the complex comorbidities that arise in the context of CVD and hepatic disease (30).

### 2.6 Endothelial dysfunction and atherosclerosis

The endothelium plays a crucial role in regulating vascular tone through the synthesis and release of various endothelium-derived relaxing factors, including vasodilator prostaglandins, nitric oxide (NO), and endothelium-dependent hyperpolarization (EDH) factors, as well as endothelium-derived contracting factors (31, 32). Endothelial dysfunction typically results from reduced production or impaired action of endothelium-derived relaxing factors and can represent an initial stage in the development of cardiovascular disease (31).

Specifically, endothelial dysfunction stands as a firmly established response to CV risk factors, taking precedence in the intricate path toward atherosclerosis. Its involvement in lesion formation encompasses a spectrum of mechanisms, ranging from the early to late stages of atherosclerosis. This involvement manifests through the up-regulation of adhesion molecules, heightened secretion of chemokines, increased adherence of leukocytes, augmented cell permeability, amplified oxidation of low-density lipoprotein, activation of platelets, production of cytokines, and the proliferation and migration of vascular smooth muscle cells. This interplay underscores the pivotal role of endothelial dysfunction in shaping the atherosclerotic landscape, illuminating the various molecular and cellular processes that contribute to atherosclerosis (33). This has led to a significant focus on evaluating endothelial function in clinical settings due to its role as an excellent surrogate marker for predicting CV events in humans. For example, impaired flow-mediated dilation of the brachial artery or a reduced digital reactive hyperemia index, as evaluated using peripheral arterial tonometry, has been linked to future CV events in individuals with coronary artery disease (34–36). These results indicate the potential value of assessing endothelial function in peripheral vascular beds as a valuable tool for predicting future CV events.

### 2.7 Inflammation

For nearly a century, cholesterol has held the position of being the primary instigator in the development of atherosclerosis. This conviction dates back to the early 1900s when researchers first identified cholesterol within arterial lesions in their experiments with animal models. This discovery has since become an indisputable testament to the role of genetic and environmental factors in contributing to atherosclerotic disease (37). In the late 1800s, inflammatory cells were first observed in atherosclerotic lesions, but only recently have we understood their crucial role in disease progression (38). This newfound understanding firmly establishes inflammation as central to both the onset and advancement of plaque formation. Endothelial injury, abnormal lipid metabolism, and hemodynamic stress are pivotal factors in early atherosclerosis. Flow-mediated inflammatory changes within endothelial cells are recognized in this process (39). Advanced atherosclerosis sees a significant influx of macrophages and inflammatory cytokines, leading to plaque destabilization and events like rupture and thrombosis (40, 41). The combined effects of proinflammatory signals within the plaque not only intensify inflammation but also impede tissue regeneration, crucial for maintaining mechanical stability (42). Proinflammatory mediators released by both immune cells and vascular endothelial cells sustain local inflammation, contributing to lesion progression (43). The revelation of the contributions made by both the innate and adaptive immune systems to atherogenesis has advanced our understanding of lesion development. This insight has also paved the way for novel therapeutic avenues aimed at alleviating the burden of vascular disease (44). However, it is crucial to acknowledge that chronic inflammatory disorders often coexist with additional metabolic comorbidities (45). In light of these complexities, a comprehensive therapeutic strategy must be devised to holistically address the cardiovascular risk in patients grappling with both chronic inflammatory disorders and associated metabolic complications.

## 3 CVD risk in psoriatic disease

The incidence of CVD is markedly elevated in individuals with inflammatory arthritis. CVD represents the primary cause of mortality in patients with psoriatic disease. Additionally, individuals with psoriatic disease exhibit a higher prevalence of traditional cardiovascular risk factors compared to the general population, exacerbating their overall cardiovascular risk (46). Patients with Pso or PsA face a higher likelihood of being overweight or obese in comparison to the general population and even when compared to individuals with other inflammatory conditions (8, 47, 48).

Studies show a strong link between these factors and NAFLD development, highlighting the pathophysiological connection between Psoriatic disease and CV comorbidities, including NAFLD. Psoriatic disease patients have a higher NAFLD prevalence than the general population (29). Additionally, the relationship between weight and Pso becomes more intriguing when abdominal obesity is considered. Multiple studies have uncovered a substantial connection between increased abdominal obesity and the presence of Pso, hinting at a possible link between weight gain and the development of this skin disorder (49). In a comprehensive 14-year study, researchers closely tracked BMI, weight changes, and central obesity indicators. Their findings revealed a persistent, strengthening link between BMI and susceptibility to PsA (50).

Moreover, PsA patients exhibit endothelial dysfunction, even without clinical evident CVD or traditional CVD risk factors (51). Furthermore, irrespective of traditional CVD risk factors, patients with PsA show a high prevalence of macrovascular disease. This is evidenced by increased carotid intima-media thickness, when compared to healthy individuals. This suggests that PsA inherently predisposes patients to cardiovascular issues beyond the influence of typical risk factors (52).

Numerous studies consistently reveal a higher prevalence of T2DM among patients with psoriatic disease compared to the general population (8, 53). In addition, individuals with PsA often present with elevated fasting glucose levels, contrasting with those suffering from RA (48). Remarkably, the severity of PsA, denoted by joint erosions, osteolysis, and sacroiliitis, has been associated with the presence of IR (54). Moreover, Pso itself is linked to an increased prevalence and incidence of T2DM, with a particularly strong correlation in severe Pso cases (55). In parallel, Psoriatic patients frequently exhibit an altered lipid profile characterized by reduced levels of high-density lipoproteins (HDL), elevated triglyceride levels (48, 53), and heightened levels of proteins associated with LDL, such as apolipoprotein B (Apo-B), when compared to healthy individuals (56). Notably, levels of Apo-B and Apo-A are positively and negatively correlated, respectively (56). In addition, some studies have proposed that PsA patients exhibit more pronounced lipid abnormalities compared to those with Pso alone (57). However, a recent investigation revealed no significant disparities between PsA and Pso patients in terms of cardiovascular risk factors. This includes metrics such as dyslipidemia (37% vs. 32%), hypertension (36% vs. 31%), T2DM (13% vs. 14%), and hyperuricemia (32% vs. 37%) (7). Interestingly, the study conducted by Husted et al. reported hypertension as the most prevalent comorbidity in PsA (37.1%), surpassing the incidence in patients with Psoriasis alone (20%). This evidence underscores the relationship between Psoriatic disease and an elevated risk of metabolic syndrome (MetS) (58). The components of MetS, such as central obesity, hypertension, IR, and dyslipidemia, are notably prevalent in PsA patients, ranging from 24% to 58% (59). MetS consistently emerges as a significant concern in various studies of PsA patients and is intricately linked to disease severity (53, 54), more than in cases of Psoriasis alone (60). Furthermore, the prevalence of MetS is notably higher in PsA than in Pso alone (61), even exceeding the prevalence in RA (47). A comprehensive review and meta-analysis found that the pooled prevalence of MetS was substantially higher in PsA compared to Pso (62).

Furthermore, systemic inflammation, as measured by C-reactive protein (CRP), has been correlated with lower levels of HDL and higher levels of triglycerides in the context of PsA (56, 63). In addition, persistent inflammation based on CRP over the previous 5 years has shown a significant association with the development of IR in PsA (56). This observation underscores the link between chronic inflammation and metabolic dysfunction in PsA, with broader implications for CVD. In PsA, inflammation arises from both skin/joint and adiposity patterns, highlighting its multifaceted nature. Adiposity also significantly influences CVD risk by contributing to a metabolic phenotype linked to increased cardiovascular complications (64).

**Figure 1:**
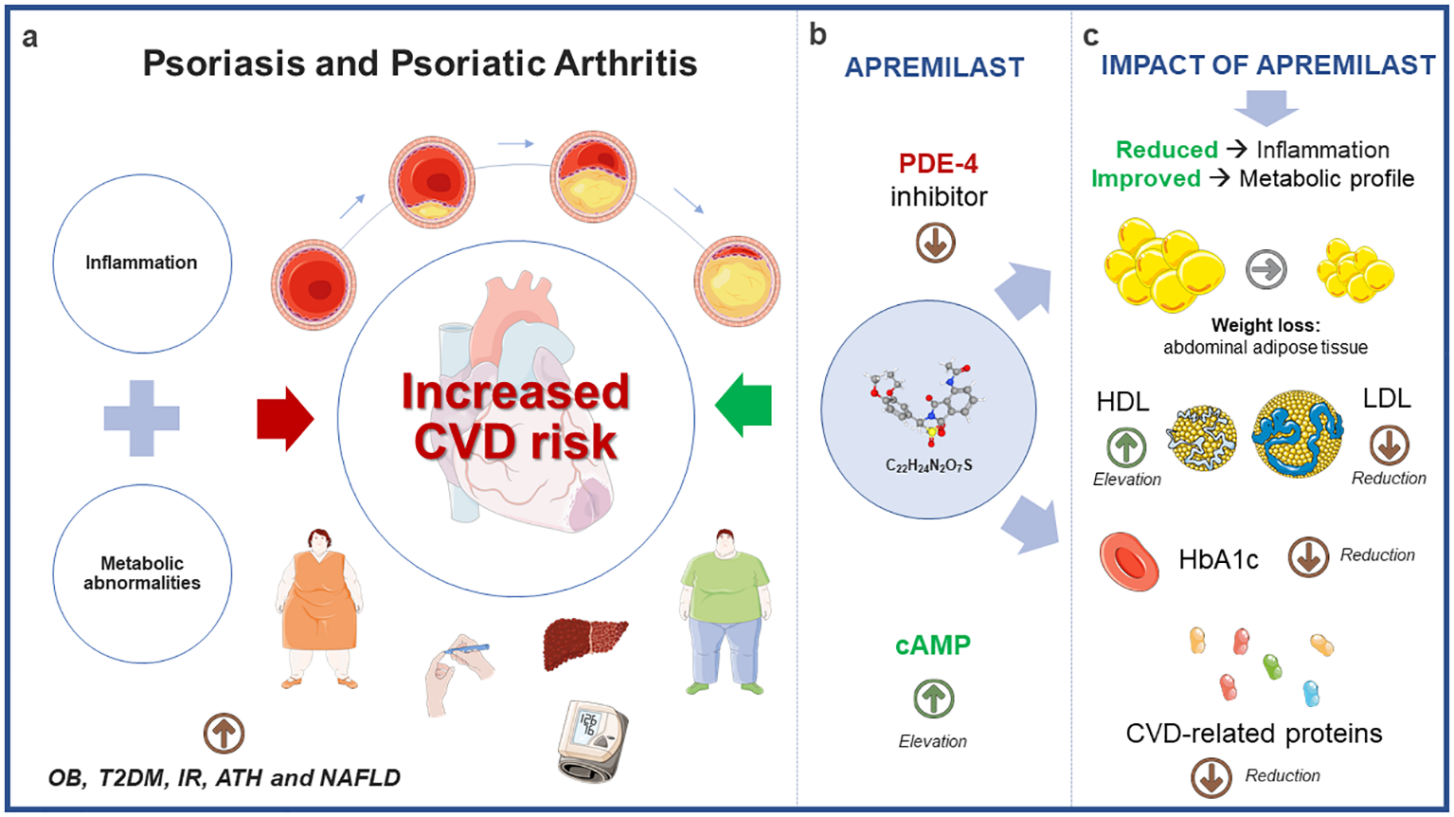
Potential impact of apremilast on cardiometabolic comorbidities associated with psoriatic disease. **(A)** The convergence of clinical characteristics in Psoriatic Disease, encompassing inflammatory patterns and metabolic irregularities, amplifies the susceptibility to cardiovascular disease. **(B)** Apremilast, by inhibiting PDE-4 and consequently elevating cAMP levels, exerts influence not only on the inflammatory profile but also on metabolic parameters. **(C)** Findings from interventional and observational studies underscore the significant effects of apremilast within the metabolic context, including weight reduction, modulation of lipid profiles, lowered HbA1c levels, and notable changes in CVD-associated proteins. Annotated names: CVD, cardiovascular disease; OB, Obesity; T2DM, Type 2 Diabetes Mellitus; IR, Insulin Resistance; ATH, Arterial Hypertension; NAFLD, Non-Alcoholic Fatty Liver Disease; PDE-4, Phosphodiesterase-4; cAMP, Cyclic Adenosine 3’, 5’-monophostate; HDL, high-density lipoprotein; LDL, low-density lipoprotein; HbA1c, Glycated hemoglobin.

The complex relationship between chronic inflammation, metabolic factors, and cardiovascular health in psoriatic disease highlights its complexity and emphasizes the necessity of a holistic approach for understanding and managing these conditions. Comprehensive care strategies should address both inflammation and metabolic health to optimize patient outcomes (**Figure 1A**).

## 4 Cyclic adenosine monophosphate and phosphodiesterase 4

One limitation of current biologic agents is their inability to directly target intracellular signaling pathways. These agents primarily act on extracellular receptors and proteins, influencing cell activity and immune signaling at the extracellular level, such as TNF inhibitors (65). However, the inability to directly target intracellular signaling pathways poses a challenge in fully intervening in complex cellular processes underlying Pso and PsA. In this sense, intracellular signaling in various cell types, including myeloid, lymphoid, and inflammatory cells, is governed by crucial “second messengers” like cAMP. The levels of intracellular cAMP are determined by the interplay between adenylylcyclases, primarily activated by G-protein coupled receptors, and phosphodiesterases (PDEs). These PDEs, expressed in a tissue-specific manner, are classified into 11 distinct families (66, 67). Interestingly, the reduction of intracellular cAMP facilitates the activity of PDE-4, resulting in an increase in inflammatory mediators and a decrease in anti-inflammatory molecules. On the contrary, inhibiting PDE-4 leads to elevated intracellular cAMP levels, which, in turn, blocks pro-inflammatory cytokines. This dynamic interplay between intracellular cAMP and PDE-4 activity plays a crucial role in modulating the inflammatory response (68).

As our comprehension of the intricate links between PDE-4 dysregulation and metabolic disorders advances, it underscores the potential importance of targeting PDE-4 as a therapeutic approach to alleviate the metabolic dysregulation and associated complications seen in T2DM and IR. The significance of PDE-4 extends to metabolic health, impacting adipocyte function, glucose regulation, hypertension, stroke, and non-alcoholic steatohepatitis. Its broad influence across metabolic domains underscores its potential as a therapeutic target for addressing metabolic disorders (69).

## 5 Effect of apremilast on the CVD profile in psoriatic disease

In this context, apremilast (C22H24N2O7S) emerges as a novel orally available small molecule that specifically targets PDE-4. Through its precise inhibition of this enzyme, apremilast effectively raises intracellular cAMP levels (**Figure 1B**). This action results in a partial suppression of several proinflammatory mediators while simultaneously promoting the production of certain anti-inflammatory mediators. Notably, these effects are more pronounced within the realm of innate immunity when compared to adaptive immunity. In addition, Apremilast boasts a low risk of serious infections and a favorable safety profile in clinical studies, offering an alternative to long-term systemic therapies, which often face issues like adverse events, safety concerns, diminishing efficacy, and injection-based administration (70).

In 2014, during the 16th week of the PALACE 1 trial, mean weight changes of -1.29 ± 3.4 kg at 20 mg and -0.97 ± 2.8 kg at 30 mg of apremilast were observed in PsA patients compared to a change of 0.19 ± 2.6 kg in the placebo group (71). Subsequently, the ESTEEM1 trial in 2015 reported a weight loss of 1.4 ± 2.08 kg in psoriasis patients, with 19% experiencing a weight loss greater than 5% (72). Around the same time, the ESTEEM2 trial noted that 20% of patients with moderate to severe plaque psoriasis exhibited a weight loss of over 5% (73). The subsequent PALACE2 trial revealed more than 5% weight loss in 17% of PsA patients on 20 mg and 14.8% on 30 mg of apremilast (74). The PALACE3 trial showed PsA patients experiencing a mean weight change of -0.05 kg (placebo), -1.2 kg (20 mg), and -1.2 kg (30 mg) (75). In 2017, a phase 2b trial in Japan revealed weight loss in 11.6% of psoriasis patients on 20 mg and 14.2% on 30 mg of apremilast (76). In 2018, the ACTIVE trial uncovered a mean weight loss of 1.20 kg at the 52-week mark in biological-naïve PsA patients, with 15.7% experiencing more than 5% weight loss (77). Additionally, the PALACE4 trial observed a higher mean BMI in PsA patients with high disease activity at week 52 (78). Recent findings from a nonrandomized clinical trial suggest that apremilast is associated with favorable impacts on cardiometabolic biomarkers and reductions in visceral and subcutaneous fat (79). These trials collectively underscore apremilast’s potential for weight reduction and broader implications for mitigating cardiovascular complications.

In 2020, Mazzilli et al. made a notable observation, showing that diabetic patients with Pso and PsA achieved superior outcomes in terms of the extent and severity of Pso when compared to their non-diabetic counterparts. The treatment not only lowered cholesterol levels in both diabetic and non-diabetic Pso/PsA patients but also led to reductions in glucose levels (80). Next, Feldmand and co-investigators compared Pso patients with and without metabolic conditions newly initiating a biological or apremilast treatment. Interestingly, nearly half of the patients discontinued their index medication over 24 months, and patients with metabolic conditions had higher discontinuation rates than those without in all treatment cohorts except apremilast (81). In addition, in our previous research, we thoroughly examined the effects of apremilast and methotrexate treatments on a cohort of PsA patients with diverse cardiometabolic profiles. Remarkably, our results unveiled that only apremilast, or the combination of apremilast and methotrexate, led to a significant reduction in disease activity among PsA patients with more prominent metabolic complications, in contrast to methotrexate monotherapy. This reduction in disease activity was not only concurrent with a decrease in body mass index but also with an improvement in IR state (56). Parallely, Ferguson et al. reported weight loss primarily in subcutaneous fat and an improvement in psoriatic disease activity, further supporting the potential benefits of apremilast in addressing PsA and related metabolic issues (82).

In a substantial cohort of patients with Pso and PsA, Orroth et al. categorized individuals into two groups based on their diabetes status: a non-diabetic group and a pre-diabetic or T2DM group. Remarkably, they observed that after six months of apremilast treatment, both weight and HbA1c levels demonstrated significant reductions, irrespective of diabetes status. Furthermore, HDL levels exhibited significant increases in patients without diabetes (83). Conversely, within this cohort of 8250 Pso/PsA patients, 26.9% were classified as obese, and 33.5% as severely obese. Interestingly, following six months of apremilast treatment, a significant reduction in weight was observed, independent of the degree of obesity. Additionally, HbA1c levels were notably reduced in Pso/PsA patients with severe obesity (84).

Data derived from the pooled analysis of five randomized, placebo-controlled, phase 3 studies, including PALACE 1-4 and ACTIVE, unveiled significant reductions in LDL cholesterol levels, particularly in patients with the highest pre-treatment levels. Additionally, this analysis indicated significant weight loss and a decrease in the rates of obesity and overweight, along with reduced HbA1c levels in patients with pre-diabetes or T2DM (85).

The SPROUT study showed that apremilast was effective and safe over 16 weeks in pediatric patients with plaque psoriasis. A significantly higher proportion achieved a Physician Global Assessment response and a ≥75% reduction in PASI scores compared to placebo, regardless of age, weight, or disease severity. Notably, younger and lower-weight patients had higher response rates (86). In addition, Guerra et al. specifically analyzed the effects of apremilast on lipid profile and weight over a one-year period. Their study demonstrated a significant reduction in weight and triglycerides at 24 and 52 weeks, as well as a significant increase in HDL levels at 52 weeks. These findings suggest that apremilast may have a positive impact on both weight management and lipid profile in individuals with moderate to severe psoriasis over the medium to long term (87). These studies represent pioneering investigations conducted in real-world clinical practice, offering valuable insights into the alterations of cardiometabolic variables following the initiation of apremilast therapy within a sizable cohort of patients suffering from Pso and PsA (**Figure 1C**). The extensive worldwide influence of apremilast on metabolic profiles is concisely outlined in **Table 1**.

**Table 1:** Impact of apremilast on the comprehensive metabolic profile of psoriatic patients.

Year	Study type	Conditions	Size	Effect of apremilast	Time	Ref.
2024	Observational: real world evidence	Pso	n=20	Weight loss, reduced triglyceride levels, and increased HDL levels.	12, 24, 52 weeks	(87)
2024	Interventional: Clinical trial (SPROUT)	Pso	n=245	Apremilast demonstrated a significantly higher rate of patients achieving PGA response and ≥75% reduction in PASI compared to placebo, irrespective of baseline age, weight, or disease severity.	16 weeks	(86)
2023	Interventional: pool of 5 clinical trial (PALACE 1-4 and ACTIVE)	Pso/PsA	N=781	Reduction in LDL cholesterol levels.	52 weeks	(85)
				Weight loss, decrease in rates of obesity and overweight.		
				Reduced HbA1c levels in pre-diabetes or T2DM patients.		
2022	Observational: real world evidence	Pso/PsA	n=8487	Weight loss.	6 months	(83)
				Reduced HbA1c levels in pre-diabetes or T2DM patients.		
				Increased HDL-cholesterol in non-diabetic patients.		
2022	Observational: real world evidence	Pso/PsA	n=8250	Weight loss independent of the degree of obesity (obese or severe obesity).	6 months	(84)
				Reduced HbA1c levels in patients with severe obesity.		
2022	Observational: real world evidence- molecular study	PsA	n=30	Decreased body mass index and insulin resistance state.	6 months	(56)
				A decrease is noted in 17 CVD-related proteins, previously identified as being altered in the plasma of PsA patients when compared to healthy donors.		
2022	Interventional: VIP-A Phase 4 clinical trial	Pso	n=70	A sustained 5% to 6% decrease in subcutaneous and visceral fat was observed after 16 weeks of treatment, and this reduction persisted through the 52-week period.	52 weeks	(79)
				Changes in cardiometabolic biomarkers related to lipoprotein characterization, inflammation and glucose metabolism.		
2022	Interventional: PALACE4 Phase 3 clinical trial	PsA	n=175	Higher body mass index at baseline in high disease activity patients.	52 weeks	(78)
2021	Interventional: IMAPA clinical trial	Pso/PsA	n=60	Weight loss, particularly the reduction of total abdominal fat, primarily targeting subcutaneous adipose tissue.	6 months	(82)
2021	Observational: real world evidence	Pso	n=7773	Less discontinuation of the treatment with apremilast in Pso patients with metabolic condition (compared to Secukinumab, Adalimumab, Ustekinumab and Etanercept).	48 months	(81)
2020	Observational: real world evidence	Pso/PsA	n=113	Lowered total cholesterol levels in both diabetic and non-diabetic Pso/PsA patients, accompanied by decreased glucose levels.	52 weeks	(80)
2018	Interventional: ACTIVE Phase 3B clinical trial	PsA	n=219	Weight loss (15.7% of patients experiencing more than 5% weight loss).	52 weeks	(77)
2017	Interventional: Japanese Phase 2B clinical trial	Pso	n=254	Weight loss:11.6% Pso patients treated with 20mg and 14.2% Pso patients treated with 30mg.	68 weeks	(76)
2016	Interventional: PALACE 3 Phase 3 clinical trial	Pso/PsA	n=505	Weight loss exceeding 5% in 14% (20mg) and 16% (30mg).	52 weeks	(75)
2016	Interventional: PALACE 2 Phase 3 clinical trial	PsA	n=484	Weight loss exceeding 5% in 17% (20mg) and 14.8% (30mg).	52 weeks	(74)
2015	Interventional: ESTEEM 2 Phase 3 clinical trial	Pso	n=413	Weight loss exceeding 5% in 20.2% of Pso patients (30mg).	52 weeks	(73)
2015	Interventional: ESTEEM 1 Phase 3 clinical trial	Pso	n=844	Weight loss exceeding 5% in 19% of Pso patients (30mg).	52 weeks	(72)
2014	Interventional: PALACE 1 Phase 3 clinical trial	PsA	n=504	In comparison to the placebo group, which exhibited a mean weight change of 0.19 ± 2.6 kg, patients with PsA demonstrated a weight change of -1.29 ± 3.4 kg at 20 mg and -0.97 ± 2.8 kg at 30 mg of apremilast.	24 weeks	(71)

Annotated names. Pso, Psoriasis; PsA, Psoriatic Arthritis; PGA, Physician Global Assessment; HDL, High Density Lipoproteins; LDL, Low Density Lipoproteins; HbA1c, glycosylated haemoglobin; T2DM, Type 2 Diabetes Mellitus; CVD, Cardiovascular Disease; PALACE, Psoriatic Arthritis Long-term Assessment of Clinical Efficacy; ACTIVE, Assessing Apremilast Monotherapy in a Clinical Trial of BIologic-NaïVE Patients With Psoriatic Arthritis; VIP-A, Vascular Inflammation in Psoriasis-Apremilast; IMAPA, The Immune Metabolic Associations in Psoriatic Arthritis; ESTEEM, The Efficacy and Safety Trial Evaluating the Effects of Apremilast in Psoriasis.

## 6 Molecular insights into apremilast impact on CVD

We identified 33 CVD-related proteins, including adipocytokines, associated with PsA at a molecular level. These proteins significantly differed in PsA patients compared to healthy controls, indicating a link between PsA and CVD characteristics. In PsA patients with varying levels of CVD comorbidities (dysregulated lipid profile, obesity, IR, hypertension, and metabolic syndrome) treated with apremilast (alone or with methotrexate), a distinct molecular profile emerged. This profile included elevated levels of proteins like CD-163, FABP-4, RARRES-2, and others in patients with multiple CVD comorbidities. Notably, this molecular profile significantly reduced with apremilast monotherapy, outperforming its combination with methotrexate or methotrexate alone after six months (week 24). These findings highlight apremilast’s potential in mitigating CVD-related markers in PsA patients with multiple metabolic comorbidities (56). Conversely, at week 16 with apremilast, favorable changes in cardiometabolic biomarkers were observed, including reductions in IL-1β, fetuin A, valine, leucine, and isoleucine. By week 52, additional improvements included reduced levels of ferritin, cholesterol efflux capacity, β-hydroxybutyrate, acetone, and ketone bodies, along with increased apolipoprotein A levels (80).

On a different note, Wang and colleagues delved into the *in vitro* effects of apremilast within the context of atherosclerosis. They employed oxidized low-density-lipoprotein (ox-LDL) to simulate the atherosclerotic microenvironment in a model of human aortic endothelial cells (HAECs). Notably, apremilast demonstrated the ability to reduce the expression of key ox-LDL scavenging receptors and inflammatory cytokines, including IL-6, TNF-α, and IL-8. In addition, apremilast effectively inhibited the attachment of monocytes to HAECs, primarily attributed to the reduction of chemokine monocyte chemotactic protein 1 (MCP-1) and the cellular adhesion molecule vascular cell adhesion molecule 1 (VCAM-1). These effects were mediated through the modulation of Krüppel-like factor 6 (KLF-6) expression, which was downregulated in response to ox-LDL via the c-Jun N-terminal Kinase (JNK) pathway (88). Besides, Otto and colleagues have presented compelling evidence regarding the potential anti-inflammatory effects of apremilast, focusing on human umbilical vein endothelial cells (HUVEC). In their study, HUVECs were exposed to TNF-α, both in the presence and absence of apremilast. Intriguingly, apremilast was found to induce a significant reduction in the secretion of pro-inflammatory mediators, including GM-CSF, CCL-2, and CXCL-10. Their investigation expanded to assess the impact of apremilast on IL-17A-induced endothelial inflammation. Significantly, apremilast effectively reduced the secretion of IL-6 and CCL-2. Additionally, apremilast demonstrated the ability to suppress adhesion molecules like VCAM-1 and E-selectin, highlighting its capacity to inhibit the adhesion of monocytic cells to activated endothelial cells, consequently impeding monocytic trans-endothelial migration. Moreover, apremilast exhibited a notable reduction in MMP-9 expression, a key player in the recruitment of inflammatory cells into the vessel wall among activated monocytic cells (89). In a recent study by Fukasawa et al., the concentrations of multiple cytokines were simultaneously and longitudinally measured at weeks 4, 16, and 24 in 20 Japanese patients with psoriasis. The study demonstrated a reduction in several serum inflammatory cytokines (IL-1β, IL-6, IL-17A, IL-17F, IL-17C, IL-21, IL-22, IL-23, IL-36γ, TGF-β1, and TNF-α) and an increase in inhibitory cytokines (IL-10 and IL-35) (90). These findings concentrate on the examination of vascular endothelial cells and present intriguing results that establish a foundation for the potential cardiovascular protective effects of apremilast treatment (**Table 2**). Nonetheless, further targeted studies would be essential to investigate the role of other metabolic cell types, such as hepatocytes or adipocytes, particularly in the context of altered metabolic conditions associated with psoriatic disease.

**Table 2:** Exploring the molecular dimensions of apremilast’s effects on CVD.

Study type	Conditions	Sample/Cell type	Effect of apremilast	Ref.
Observational: real world evidence- molecular study	PsA patients	Blood-based biomarkers: circulating CVD-plasma levels	A decrease is noted in CD-163, FABP-4, RARRES-2, CCL-15, MMP-3, vWF, GDF-15, TPA, TIMP-4, TR-AP, IL-2RA, CTSD, CNTN-1, GAL-3, LTBR, OPG and NT-proBNP at 6 months, previously identified as being altered in the plasma of PsA patients when compared to healthy donors.	(56)
Interventional: VIP-A Phase 4 clinical trial	Pso patients	Blood-based biomarkers: circulating plasma and serum levels	Changes in cardiometabolic biomarkers related to lipoprotein characterization, inflammation and glucose metabolism.	(79)
			At week 16, there was a reduction in IL-1β, valine, leucine, and feutin A, along with a decrease in branched-chain amino acids. By week 52, a decline was observed in ferritin, β-hydroxybutyrate, acetone, and ketone bodies, accompanied by an increase in apolipoprotein A-1.	
Molecular study	*In vitro:* gene and protein expression	Human aortic endothelial cells (HAECs) and U937 monocytic cells	Apremilast exhibited the capacity to decrease the expression of crucial receptors involved in oxidized ox-LDL scavenging, along with a reduction in inflammatory cytokines such as IL-6, TNF-α, and IL-8 upon stimulation with ox-LDL.	(88)
			Apremilast successfully hindered the attachment of monocytes to HAECs, primarily due to the diminished levels of MCP-1 and VCAM-1.	
Molecular study	*In vitro*: *gene and protein expression*	Human umbilical vein endothelial cells (HUVEC) and THP1 monocytic cells	Apremilast effectively inhibited the TNF-α-induced expression and secretion of crucial pro-inflammatory factors in both endothelial and monocytic cells. These factors encompass GM-CSF, CXCL-10, CCL-2, VCAM-1, E-selectin, and MMP-9.	(89)
			Apremilast diminished the adhesion of THP-1 cells to activated HUVECs and the Transwell Endothelial Migration in response to TNF-α.	
			Apremilast inhibited the activation of NFκB and MAPK signaling in activated HUVECs.	
			Apremilast decreased IL-17A-induced secretion of IL-6 and CCL2.	
Observational: real world evidence- molecular study	Pso patients	Blood-based biomarkers	Decreased levels of IL-1β, IL-6, IL-17A, IL-17F, IL-17C, IL-21, IL-22, IL-23, IL-36γ, TGF-β1, and TNF-α, alongside an elevation in inhibitory cytokines such as IL-10 and IL-35.	(90)

Annotated names. CVD, Cardiovascular Disease; PsA, Psoriatic Arthritis; Pso, Psoriasis; VIP-A, Vascular Inflammation in Psoriasis-Apremilast; CD-163, Cluster Differentiation 163; FABP-4, Fatty Acid Binding Protein 4; RARRES-2, Retinoic Acid Receptor Responder 2; CCL-15, C-C Motif Chemokine Ligand 15; MMP-3, Matrix Metallopeptidase 3; vWF, Von Willebrand Factor; GDF-15, Growth Differentiation Factor 15; TPA, Tissue Type Plasminogen Activator; TIMP-4, Tissue Inhibitor of Metalloproteinase; TR-AP, Tumor necrosis factor (TNF) receptor associated factor 2; IL-2RA, Interleukin 2 Receptor Subunit Alpha; CTSD, Cathepsin D; CNTN-1, Contactin 1; GAL-3, Galectin 3, LTBR, Lymphotoxin Beta Receptor; OPG, Osteoprotegerin; NT-proBNP, N-Terminal Prohormone Recognition Protein 1; IL-1β, Interleukin 1-beta; LDL, Low Density Lipoprotein; IL-8, Interleukin 8; MCP-1, Monocyte Chemotactic Protein 1; VCAM-1, Vascular Cell Adhesion Molecule 1; GM-CSF, Granulocyte-Macrophage Colony Stimulating Factor; CXCL-10, C-X-C Motif Chemokine Ligand 10; CCL-2, C-C Motif Chemokine Ligand 2; MMP-9, Matrix Metallopeptidase 9; NFκB, Nuclear Factor Kappa Beta; MAPK, Mitogen-Activated Protein Kinase; IL-17A, Interleukin 17A; IL-6, Interleukin 6.

## 7 Conclusions

1. Psoriatic disease links strongly to higher cardiovascular risk, including type 2 diabetes, obesity, IR, hypertension, and dysregulated lipids, raising mortality risk.

2. Given the increased cardiometabolic risk in psoriatic disease, treatment focus must shift to manage metabolic factors alongside inflammation.

3. Apremilast, with its dual-action potential, emerges as a pivotal treatment for psoriatic disease, addressing both inflammation and metabolic parameters, enhancing overall well-being.

## Author contributions

NB: Conceptualization, Funding acquisition, Methodology, Supervision, Writing – original draft, Writing – review & editing. CL-M: Methodology, Supervision, Writing – review & editing. AE-C: Methodology, Supervision, Writing – review & editing. IA-dR: Conceptualization, Methodology, Writing – original draft, Writing – review & editing.
